# Quality of life of a healthy polish population due to sociodemographic factors during the COVID-19 pandemic – a cross-sectional study

**DOI:** 10.3389/fpubh.2023.1204109

**Published:** 2023-08-17

**Authors:** Marlena Krawczyk-Suszek, Andrzej Kleinrok

**Affiliations:** ^1^Department of Physiotherapy, Medical College, University of Information Technology and Management in Rzeszow, Rzeszów, Poland; ^2^Institute of Humanities and Medicine, Academy of Zamosc, Zamosc, Poland

**Keywords:** SF-36, health, sociodemographic factors, smoking, physical activity

## Abstract

**Introduction:**

The quality of life should be studied in every person, both among the sick and healthy. Sociodemographic factors affect the level of the perceived quality of life (QoL), and especially in the situation of the COVID-19 pandemic, which forced the enforcement of certain behaviours in society, such as social distancing, as well as introduced panic and fear for one’s own health and life. The main aim of the study was to assess the quality of life in the group of people without the disease, to assess the impact of sociodemographic factors on QoL during the pandemic.

**Material and method:**

3,511 healthy people were included in the study. The inclusion criteria of the study were: age of respondents over 18 years, no continuously administered medicaments, no diagnosed chronic diseases and no treatment in specialist clinics as well as lack of positive COVID-19 test in 4 weeks before the examination. The SF-36 questionnaire was used to assess the quality of life. The student’s *t*-test and intergroup comparisons were used in 7 age groups. Factors such as age, gender, place of residence, education, civil status, employment status, smoking, and physical activity were assessed.

**Results:**

The lowest average QoL level in the studied population was recorded in the Mental Component Summary (MCS) dimension (*X* = 47.9;Cl:47.6–48.3). A high correlation between age and the SF-36 spheres was noted in the following spheres: physical functioning (PF), role physical (RP), Physical Component Summary (PCS), and ILQ (*p* < 0.001). The highest chance of a better QoL in the PCS dimension among men was recorded in the 30–39 age group (OR = 3.65;Cl:1.13–11.79). In the group of people over 50 years of age living in the village, there was a greater chance of a better QoL in the PCS dimension in each age group. Practicing physical activity was significantly more often conditioned by a higher chance of developing a better QoL (*p* < 0.05). In the group of people ≥80 years of age, there was a greater than 4 times higher chance of developing a better quality of life in terms of MCS among physically active people (OR = 4.38;Cl:1.62–11.83).

**Conclusion:**

With age, QoL decreases among people with disabilities. Men are more likely to assess their health better. A better QoL among women occurs at age 80 and later. A higher level of education often determined a significantly higher level of QoL felt. The practising of recreational physical activity and the lack of smoking habit determined a higher level of QoL more often. Smoking provided a greater chance of a better QoL in ILQ in the group of people ≥80 years.

## 1. Introduction

### 1.1. The concept of quality of life in the health population

Quality of life (QoL) encompasses different aspects of an individual’s life and is interpreted differently by a number of experts. QoL is a subjective assessment of one’s own life position, with particular emphasis on the context of the individual’s views, i.e., values, interests, expectations and external factors (1).

Poor human health will also affect the family and other people living in close proximity to a person. Such a condition may lead to a decrease in the quality of life related to health (HRQoL) and even shortened life. Analysing the state of the community, the reduced quality of life will hinder economic and social development, which may affect the reduction of human capital and its potential. On the other hand, a long and healthy life can be an important indicator of an individual’s well-being, but it can also confirm social success in a holistic way (2).

The literature on the subject includes the entire spectrum of research on the quality of life of people with various diseases (3–5). There are few scientific reports on the population of healthy people and the assessment of quality of life in each age group. However, this analysis can have a significant value for QoL comparisons between healthy and sick people.

At the present time, the quality of life of healthy people, but functioning in reality with the COVID-19 pandemic, may differ slightly from the quality of life of healthy people before the pandemic, especially in such aspects of everyday life as social distancing, isolation and fundamental changes in the routine of everyday activities, which may condition changes in the physical and mental dimension of QoL (6).

### 1.2. Quality of life and factors determining it

Health referred to as “a state of complete physical, mental and social well-being and not merely the absence of disease or infirmity” (7) will always be a multi-pronged concept for which it will be impossible to find a single indicator defining QoL.

HRQoL is influenced by health, mental state, independence from the environment and other people, social connections (8). In the literature, the importance of many QoL measures detrimental to the health of adolescents is found, but groups burdened with disease units are included in the study (9–11).

The health status and associated HRQoL changes with age, especially in later life (12). Although often despite biological changes in the body, the older adults maintain good physical and psychological health (13). Demographic projections constantly highlight the continuing trend of an ageing population (14), which will generate many social and economic problems for states. Based on the information contained in the Eurostat report, numerous attempts were made to estimate the factors significantly affecting the quality of life, including demographic, socio-economic factors, economic and social transformations, together with the analysis of the gross domestic product (GDP) (15).

Stressful events in life can cause disruption to the overall QoL. In the light of the COVID pandemic, working conditions, especially in the case of health care professionals, influenced the sense of quality of life, which also results from the sense of security of the individual, including health security (16). Other global studies show that during the outbreak of the pandemic, there is a deterioration in the overall QoL, especially in the group of women with poor health, which confirms the importance of gender in the sense of HRQoL (17). The outbreak of the SARS-CoV-2 pandemic, known as COVID-19, occurred in 2019 (18, 19) and due to the high reproduction rate (R0 = 3,84) (20) the virus spread very quickly to all countries of the world. The number of infections and complications caused a general panic among the population, especially in the group of infected people, as well as in the group of people who are at risk of infection (21, 22). The introduction of social distancing by individual countries, disruption of the rhythm of life and performance of everyday activities, reduction of employment and thus income, and the introduction of restrictions related to the provision of medical services and the introduction of only forms of telemedicine in a large percentage (23) resulted in the appearance of negative emotions directly affecting the psychological sphere of a person. This situation defines the need to assess the quality of life of healthy people functioning in a different reality than before.

Changing factors that will be important elements of everyday life, such as freedom of movement and interpersonal contacts severely limited during the pandemic, currently returning to normal situation, information related to threats such as war, individual factors such as gender, place of residence or workplace (24) determine the strongly felt level of the QoL in healthy people. For many years, there have been indications in the literature that prolonged exposure to factors such as stressors causes changes in the sense of health, especially in the mental sphere (25) or a preference for stimulants such as smoking. In a long-term analysis, this contributes to a change in health and the QoL. Initially, behavioural, psychological and medical problems appear, such as alcohol abuse or smoking, and as a consequence – full-blown stress (26). At this point, the health promotion emphasized by many specialists is an important element of the country’s health policy, which must include the analysis of risk factors in a group of healthy people and the observation of the strength of the correlation of these factors with the level of the QoL, which is why the analysis of the level of the QoL in this group of people is very important.

The aim of the study was to assess the quality of life (QoL) of healthy people in the Polish population during the COVID-19 pandemic, to analyze the impact of selected sociodemographic factors on higher QoL and to assess the SF-36 dimensions/sphere, taking into account age groups. The following factors were included in the analysis: gender, place of residence, education, employment status, marital status, smoking and physical activity.

## 2. Materials and methods

### 2.1. Organization of the study

The study was carried out randomly among healthy people in the Polish population. The study was conducted on a group of 5,076 healthy people, who were over the age of 18.

**Figure 1:**
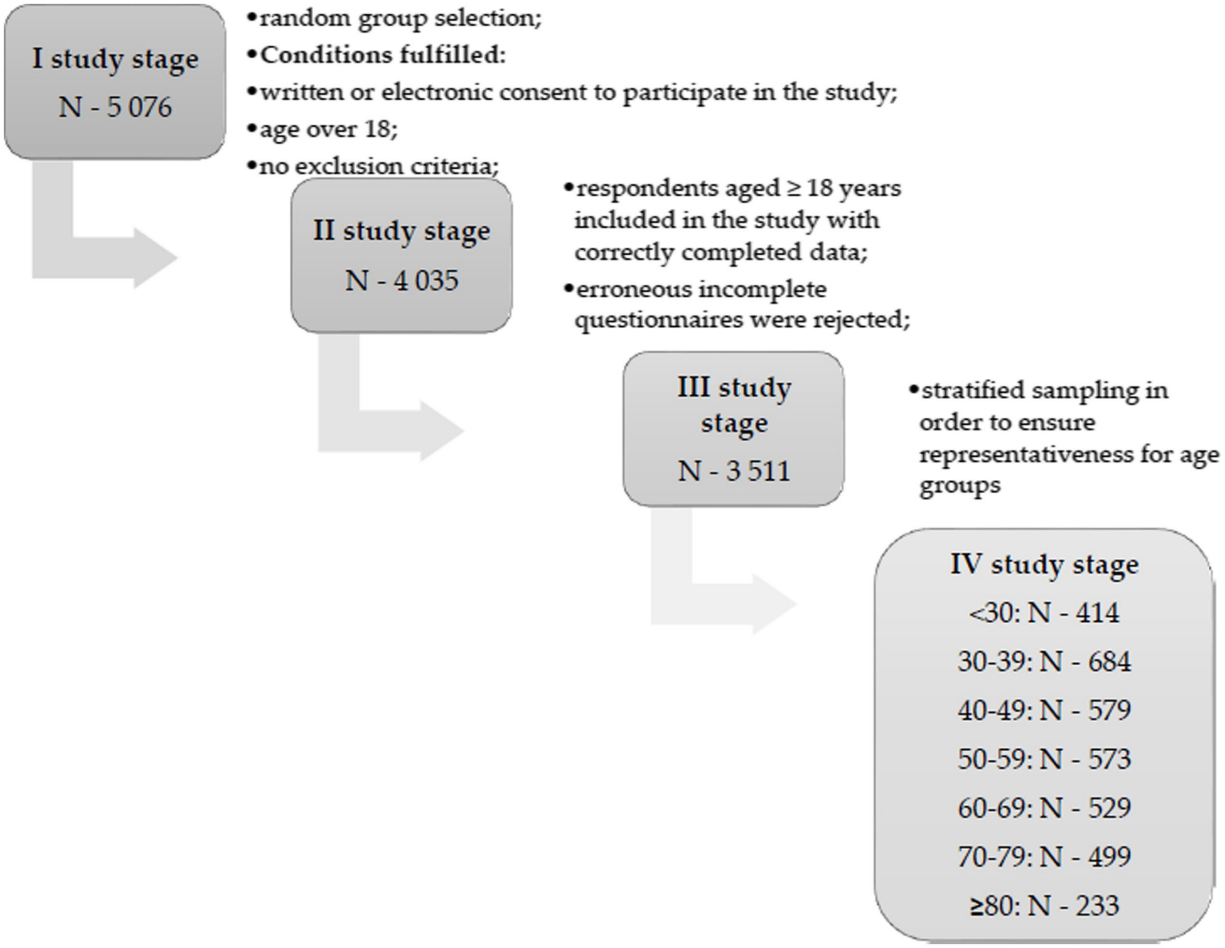
Flow chart of the participants selection process.

The inclusion criteria were: age of respondents over 18 years, no constantly administered medicaments, no diagnosed chronic diseases and no treatment in specialist clinics as well as lack of positive COVID-19 test in 4 weeks before examination. The absence of COVID-19 infection in the last 4 weeks allows to reduce the risk of infection impact on the perceived quality of life of the subjects. Stratified random selection was applied sequentially to ensure the representativeness of the sample in all analysed age groups. Finally, the study was conducted among 3,511 healthy people. They completed the survey and the SF-36 questionnaire. A detailed flow chart is presented in Figure 1. In the “Information for the respondent” before the start of the study, the respondents were informed about the anonymity, lack of risk for the respondents related to participation in the study and the possibility of withdrawing from participation in the study at every stage. Before participating in the study, the respondents provided their informed consent to participate in the study (in paper or electronic form).

The test strength for the Mi1 = Mi2 hypothesis (mean with respect to gender) was assessed assuming Es = 0.25 and *p* ≤ 0.05. The strength test was 1.00.

A positive opinion was obtained from the Commission on Ethics of Scientific Research of the University of Information Technology and Management in Rzeszow (2/2022). The study was conducted in accordance with the Declaration of Helsinki Ethical Principles for Medical Research Involving Human Subjects (27).

### 2.2. Study group

Finally, a group of 3,511 people were included in the study. The study participated by 1754 women (50,0%) and 1757 men (50,0%). The average age of the respondents was 51,2 ± 17,3 years old. The structure of the distinguished age groups corresponds to the indicators of the Central Statistical Office (28). The characteristics of the examined group of healthy people are presented in Table 1.

**Table 1:** General characteristics of the study group of the respondents.

Characteristics	Age group [*n*;%]														Total [*n*;%] [3.511; 100.0]	
	<30 [414; 11.8]		30–39 [684; 19.5]		40–49 [579; 16.5]		50–59 [573; 16.3]		60–69 [529; 15.1]		70–79 [499; 14.2]		≥80 [233; 6.6]			
	*n*	%	*n*	%	*n*	%	*n*	%	*n*	%	*n*	%	*n*	%	*n*	%
Gender:-female/male	133/281	32.1/67.9	324/360	47.4/52.6	305/274	52.7/47.3	271/302	47.3/52.7	255/274	48.2/51.8	300/199	60.1/39.9	166/67	71.2/28.8	1754/1757	50.0/50.0
Place of residence-city/village	250/164	60.4/39.6	417/267	61.0/39.0	317/262	54.7/45.2	322/251	56.2/43.8	280/249	52.9/47.1	275/224	55.1/44.9	123/110	52.8/47.2	1984/1.527	56.5/43.5
Education-higher/secondary/elementary	234/167/13	56.5/40.4/3.1	395/265/24	57.7/38.7/3.5	280/269/30	48.4/46.5/5.2	205/316/52	35.8/55.1/9.1	115/311/103	21.7/58.8/19.5	73/233/193	14.6/46.7/38.7	23/92/118	9.9/39.5/50.6	1.325/1.653/533	37.7/47.1/15.2
Employment status-works/disability pension/retirement/does not work	358/0/0/56	86.5/0.0/0.0/13.5	645/0/0/39	94.3/0.0/0.0/5.7	517/0.0/0.0/62	89.3/0.0/0.0/10.7	472/33/31/37	82.4/5.8/5.4/6.4	212/53/222/42	40.1/10.0/42.0/7.9	7/31/450/11	1.4/6.2/90.2/2.2	0/7/217/9	0.0/3.0/93.1/3.9	2.211/124/920/256	63.0/3.5/26.2/7.3
Partnership- in relationship/single	141/273	34.1/65.9	244/240	64.9/35.1	494/85	85.3/14.7	465/104	81.1/18.1	419/110	79.2/20.8	347/152	69.5/30.5	123/110	52.8/47.2	2.433/1.078	69.3/30.7
Smoking yes/no	108/306	26.1/73.9	173/511	25.3/74.7	128/451	22.1/77.9	127/446	22.2/77.8	83/446	15.7/84.3	69/430	13.8/86.2	25/208	10.7/89.3	713/2.798	20.3/79.7
Physical Activityyes/no	223/191	53.9/46.1	367/317	53.6/46.3	245/334	42.3/57.7	228/345	39.8/60.2	188/341	35.5/64.5	130/369	26.0/74.0	21/212	9.0/91.0	1.402/2.109	39.9/60.1

### 2.3. Questionnaire SF-36 and sociodemographic factors

The study used an own structure questionnaire and the standardized SF – 36v.2 questionnaire to assess the quality of life. The SF-36 quality of life questionnaire is still the most frequently used tool for carrying out the quality of life study in both healthy and sick people, as a reliable tool for the study (29, 30). The SF – 36v.2 quality of life questionnaire allows to assess the quality of life in the following dimensions: physical functioning (PF), role physical (RP), bodily pain (BP), general health (GH), vitality (VT), social functioning (SF), role emotional (RE), and mental health (MH) (31).

The analysis of individual dimensions was performed in accordance with the tool key. The individual parameters were combined into two groups, including four parameters regarding the assessment of the physical sphere and four parameters covering the mental sphere. The spheres were assigned as follows: to the PCS dimension (Physical Component Summary) the following spheres were assigned: PF + RP + BT + GH; to the MCS dimension (Mental Component Summary) the following spheres were assigned: VT + SF + RE + MH. Both indicated dimensions form the Quality of Life Index (ILQ) (31).

A license was obtained to use the SF-36 questionnaire (License Number: QM039882).

The SF-36 tool was used to analyse quality of life (QoL) in a group of healthy people. The conducted analysis included the assessment of the reliability of a standardized tool for assessing the analyzed feature – QoL. For the measured variables, mean point values and standard deviation with confidence interval were calculated, as well as the range of values and the upper and lower quartile for individual spheres and dimensions. The value of Cronbach’s alpha (*α*) reliability coefficient was assessed, evaluating the correlations of variables in their own dimension (internal consistency). The value of the coefficient for the analyzed SF-36 variables was above 0.78. A satisfactory value of the Alpha-Cronbach coefficient indicates a high homogeneity of the SF-36 questionnaire used in the study. Cronbach’s *α* is a cumulative measure. The coefficient takes a value from 0 to 1, values above 0.7 are considered acceptable (32).

In the original version of questionnaire SF-36, **Question 1** is coding the following percentages: 1 – Excellent – 100%; 2 – Very good – 75%; 3 – Good – 50%; 4 – Fair – 25%; 5 – Poor – 0%. The following coding method: 1 = 100%, 2 = 75%, 3 = 50%, 4 = 25%, 5 = 0% applies to questions: **1,2,20,22,34,36**. The following coding method: 1 = 0%, 2 = 50%, 3 = 100% applies to questions: **3,4,5,6,7,8,9,10,11,12**. The following coding method: 1 = 0%, 2 = 100% applies to questions: **13,14,15,16,17,18,19**. The following coding method: 1 = 100%, 2 = 80%, 3 = 60%, 4 = 40%, 5 = 20%, 6 = 0% applies to questions: **21,23,26,27,30**. The following coding method: 1 = 0%, 2 = 20%, 3 = 40%, 4 = 60%, 5 = 80%, 6 = 100% applies to questions: **24,25,28,29,31**. The following coding method: 1 = 0%, 2 = 25%, 3 = 50%, 4 = 75%, 5 = 100% applies to questions: **32,33,35** (33). For details see questionnaire.

The analysis assessed the following dimensions: PCS, MCS, and ILQ. Some authors argue that the overall quality of life index should not be verified and assessed, which is a mistake. However, it seems that showing the value of the overall quality of life index (ILQ) provides an overall assessment of the quality of life, which, as is known, is not a factor that can be examined as one dimension. Therefore the ILQ indicator is only a complement to the analyses carried out in the area of MCS and PCS dimensions. We present detailed and general indicators without deciding on their significance (Table 2).

**Table 2:** Reliability of the SF-36 questionnaire in the examined Polish group of healthy people and the distribution of the values of variables for the total number of respondents.

Spheres	*X* (−95Cl − +95Cl)	SD (−95Cl − +95Cl)	Me	Q1	Q3	Cronbach’s alpha (*α*)
PF	83.9 (83.2-84.7)	22.0 (21.5-22.6)	95.0	75.0	100.0	0.80
RP	63.2 (61.8-64.6)	41.8 (40.8-42.8)	75.0	25.0	100.0	0.80
RE	67.6 (66.3-68.9)	40.7 (39.7-41.6)	100.0	33.3	100.0	0.81
VT	60.7 (60.4-61.0)	8.0 (7.8-8.2)	60.0	55.0	65.0	0.84
MH	45.6 (45.3-45.8)	7.5 (7.3-7.6)	46.7	40.0	50.0	0.85
SF	71.2 (70.4-72.0)	24.4 (23.9-25.0)	75.0	50.0	100.0	0.82
BP	70.6 (69.8-71.4)	23.6 (23.1-24.2)	67.5	55.0	90.0	0.80
GH	54.0 (53.7-54.4)	11.6 (11.3-11.9)	55.0	45.0	60.0	0.83
MCS	47.9 (47.6-48.3)	10.6 (10.4-10.9)	52.5	38.8	56.3	0.81
PCS	67.9 (67.3-68.6)	18.9 (18.5-19.4)	72.5	53.8	83.8	0.78
ILQ	57.9 (57.5-58.4)	13.4 (13.1-13.7)	61.6	47.5	69.0	0.79

*X, average; −95Cl/+95Cl, 95% confidence interaval of average or standard deviation; SD, standard deviation; Me, median; Q1, lower quartile; Q3, upper quartile; PF, physical functioning; RP, role physical; RE, role emotional; VT, vitality; MH, mental health; SF, social functioning; BP, bodility pain; GH, general health; MCS, mental component summary; PCS, physical component summary; ILQ SF-36, index of life quality.

The following levels of variables were included in the analysis. The “partnership” variable was a dichotomous variable that was assigned two levels: it would remain in a relationship and it would remain single. Staying in a relationship meant staying married or in a partnership, while staying single meant not having a relationship with the other person. The variable “smoking” was also a dichotomous variable, the possible answers were: yes-no. The analysis of smoking in this study did not include passive smokers. Another variable, “physical activity,” was also a dichotomous variable with yes-no levels. Physical activity was assessed on the basis of the definitions and recommendations indicated by the WHO. According to the recommendations published in 2020, physical (recreational) activity in the group of adults between 18 and 64 years of age should last at least 150–300 min per week of moderate intensity or 75–150 min of high-intensity physical activity. Adults should do at least 2 exercises a week to strengthen the main muscle groups. In the case of people over 65 years of age, caution should be exercised, before practising physical activity, consult a doctor. However, training in this group of people should be focused on exercises of various nature with moderate or greater intensity, placing particular emphasis on balance and muscle strengthening. The WHO recommendations assume that this type of exercise should be performed at least 3 times a week in order to effectively reduce the risk of falls (34). Fulfillment of the above assumptions by the respondents, in different age groups, was tantamount to giving an affirmative answer regarding physical activity.

### 2.4. Statistical analysis

Only reliable data were included in the analysis, missing data were excluded from the proper analysis.

In the conducted analysis, multiple comparisons were used to present the *p*-value for comparisons of the levels of the age groups variable in spheres and dimensions of SF-36 questionnaire. For the purposes of the study and data analysis, the study group was divided into 7 age groups: <30 years old, 30–39 years old, 40–49 years old, 50–59 years, 60–69 years, 70–79 years, and ≥80 years. The Student’s *t*-test was used.

Measurable variables were presented using: mean values, 95% of the range for the mean value, standard deviation (SD), 95% of the range for the SD value, median, upper quartile and lower quartile values. The Alpha Cronbach coefficient was used to assess the reliability of the SF-36 tool. With a view to indicate the absolute value of the differences for the mean values of the two analysed variables, the following formula was used: Ix¯1−x¯2I , and for several analysed variables, the absolute value of the difference between the highest and lowest mean, according to the formula: I $\bar{x}$ _MAX_ − $\bar{x}$ _MIN_I.

The analysis of the linear correlation between measurable variables was carried out using the Pearson’s *r* coefficient. The following scale was adopted to assess the degree of dependence of two variables: 0–0.3 – weak correlation; 0.3–0.5 – average correlation; 05–0.7 – high correlation; 0.7–0.9 – very high correlation, and 0.9–1 – almost full correlation. The coefficient sign means: a positive correlation, in which an increase in one variable is observed in the other, or a negative correlation, in which an increase in one variable causes a decrease in the other. The absolute value of the coefficient confirms the strength of the relationship of the analysed variables. Spearman’s rho coefficient was used to assess the correlation between qualitative and measurable variables (35).

A logistic regression was used in the analysis. The chances of a higher quality of life were assessed, taking into account the analyzed factors. The odds ratio (OR) and the 95% confidence interval (−95% CL; +95Cl) were presented. For the purposes of the analysis, the measurable variables: MCS, PCS, and ILQ were recoded to the dichotomous variable dimension. The analyzed quality of life levels were marked at 50% and below, evaluating these values as low QoL, and quality of life above 50% as high QoL.

The analysis was carried out using the statistical program Statistica 13.0.

## 3. Results

The analysis presents the mean and standard deviations of the percentages obtained in the studied age groups in all spheres and dimensions of the SF-36 questionnaire.

The average percentages in the analysed groups were the highest in the group of 30–39 years and in the case of most of the spheres and dimensions of SF-36 they decreased in subsequent older age groups. The detailed data are presented in Table 3.

**Table 3:** Average level of the QoL in individual spheres, considering the intergroup averages.

Variable	Spheres										
	PF	RP	RE	VT	MH	SF	BP	GH	MCS	PCS	ILQ
<30 [*n* = 414]											
X	96.5	82.1	83.0	60.9	54.3	79.0	83.5	58.0	76.6	72.9	74.8
−95Cl	95.8	79.0	80.4	60.2	53.4	76.7	81.7	56.8	75.2	72.1	73.7
+95Cl	97.1	85.2	85.7	61.5	55.1	81.3	85.2	59.1	78.0	73.7	75.8
SD	6.8	32.3	27.4	6.8	9.0	23.8	18.0	12.0	14.3	8.4	10.4
−95Cl	6.4	30.3	25.7	6.4	8.4	22.3	16.9	11.2	13.4	7.8	9.7
+95Cl	7.3	34.7	29.4	7.3	9.7	25.6	19.3	12.8	15.4	9.0	11.2
Me	100.0	100.0	100.0	60.0	56.0	88.0	90.0	60.0	81.0	74.0	78.0
30–39 [*n* = 684]											
X	96.8	85.1	86.7	61.1	51.9	80.5	84.0	56.2	78.1	72.5	75.3
−95Cl	96.2	82.9	84.6	60.6	51.2	78.8	82.6	55.3	77.1	71.9	74.6
+95Cl	97.4	87.2	88.8	61.7	52.6	82.2	85.4	57.1	79.1	73.1	76.0
SD	8.3	28.0	27.7	7.4	9.2	22.3	18.6	12.0	13.3	8.5	9.8
−95Cl	7.8	26.6	26.2	7.1	8.8	21.2	17.7	11.4	12.7	8.0	9.3
+95Cl	8.7	29.6	29.1	7.8	9.7	23.5	19.6	12.7	14.1	8.9	10.3
Me	100.0	100.0	100.0	60.0	52.0	87.5	90.0	60.0	82.5	75.0	78.7
40–49 [*n* = 579]											
X	92.0	75.6	76.2	60.2	53.5	73.8	75.8	55.2	71.5	69.1	70.3
−95Cl	91.1	72.6	73.2	59.4	52.7	71.8	74.1	54.3	70.2	68.2	69.3
+95Cl	93.0	78.6	79.1	61.0	54.3	75.7	77.5	56.1	72.8	70.0	71.3
SD	12.2	36.3	35.8	9.6	10.0	24.2	20.6	11.0	15.7	10.9	12.2
−95Cl	11.5	34.3	33.9	9.1	9.4	22.9	19.5	10.4	14.9	10.3	11.5
+95Cl	13.0	38.5	38.0	10.2	10.6	25.7	21.9	11.6	16.7	11.6	12.9
Me	95.0	100.0	100.0	60.0	56.0	75.0	78.0	55.0	76.0	73.0	75.0
50–59 [*n* = 573]											
X	87.7	67.0	70.8	61.7	54.4	73.2	69.6	54.5	68.8	65.9	67.4
−95Cl	86.3	63.7	67.7	61.0	53.7	71.3	67.8	53.6	67.5	64.8	66.2
+95Cl	89.0	70.2	73.9	62.4	55.2	75.1	71.5	55.4	70.2	67.0	68.5
SD	16.4	39.5	38.3	8.5	8.8	23.2	22.2	10.8	16.5	12.9	13.6
−95Cl	15.5	37.4	36.2	8.0	8.4	21.9	21.0	10.2	15.6	12.2	12.9
+95Cl	17.4	41.9	40.6	9.0	9.4	24.6	23.6	11.4	17.6	13.7	14.5
Me	95.0	75.0	100.0	60.0	56.0	75.0	68.0	55.0	72.3	71.3	71.9
60–69 [*n* = 529]											
X	78.2	47.8	55.8	60.5	56.1	66.0	62.7	53.8	61.3	59.0	60.1
−95Cl	76.5	44.2	52.1	59.9	55.4	64.1	61.0	52.9	59.8	57.9	59.0
+95Cl	79.9	51.4	59.6	61.1	56.7	68.0	64.4	54.8	62.7	60.1	61.3
SD	19.7	42.1	44.0	7.4	8.1	22.5	19.7	11.1	16.9	13.1	13.7
−95Cl	18.6	39.7	41.5	7.0	7.6	21.2	18.6	10.5	15.9	12.3	12.9
+95Cl	21.0	44.8	46.8	7.9	8.6	23.9	21.0	11.8	18.0	13.9	14.5
Me	85.0	50.0	66.7	60.0	56.0	62.5	65.0	55.0	62.3	58.5	61.3
70–79 [*n* = 499]											
X	64.0	35.6	43.8	60.4	57.7	59.5	56.2	49.4	55.0	51.7	53.3
−95Cl	61.9	32.0	39.9	59.8	57.0	57.5	54.2	48.4	53.3	50.3	51.9
+95Cl	66.2	39.2	47.6	61.1	58.4	61.5	58.2	50.3	56.6	53.0	54.7
SD	24.4	40.9	44.0	7.4	7.6	22.7	22.7	10.9	18.8	14.9	15.7
−95Cl	23.0	38.5	41.4	7.0	7.2	21.4	21.4	10.3	17.7	14.1	14.8
+95Cl	26.0	43.6	46.9	7.9	8.1	24.2	24.3	11.7	20.0	15.9	16.8
Me	65.0	25.0	33.3	60.0	60.0	62.5	57.5	50.0	51.9	50.5	52.7
≥80 [*n* = 233]											
X	50.2	15.7	34.2	58.9	57.5	54.9	46.7	47.1	48.7	42.6	45.6
−95Cl	46.5	12.0	28.8	57.9	56.6	52.0	43.8	45.9	46.5	40.9	43.9
+95Cl	53.9	19.3	39.6	59.9	58.3	57.7	49.6	48.4	50.8	44.3	47.4
SD	28.7	28.2	41.7	7.5	6.6	22.2	22.3	9.7	17.0	13.4	13.8
−95Cl	26.3	25.8	38.3	6.9	6.1	20.3	20.5	8.9	15.5	12.2	12.7
+95Cl	31.6	31.0	45.9	8.3	7.3	24.4	24.6	10.7	18.7	14.7	15.2
Me	50.0	0.0	0.0	60.0	56.0	50.0	45.0	45.0	44.4	39.0	42.0

*X, average; −95Cl/+95Cl, 95% confidence interaval of average or standard deviation; SD, standard deviation; Me, median; PF, physical functioning; RP, role physical; RE, role emotional; VT, vitality; MH, mental health; SF, social functioning; BP, bodility pain; GH, general health; MCS, mental component summary; PCS, physical component summary; ILQ SF-36, index of life quality.

The impact of sociodemographic variables on QoL was assessed, taking into account the following dimensions: PCS, MCS, and ILQ. According to the tool’s key, the higher the percentage of points obtained in individual dimensions, the better the QoL.

In the group of people up to 30 years of age, women significantly more often indicated a better quality of life in the PCS dimension (74.1 ± 6.6; *p* = 0.049). The place of residence significantly differentiated the level of quality of life in each of the dimensions (*p* < 0.001). A better quality of life in terms of PCS, as well as in MCS and ILQ was recorded in people living in the countryside (respectively: PCS:74.7 ± 6.4; MCS:80.1 ± 13.5; ILQ:77.4 ± 8.7). A significantly better QoL in the PCS and ILQ dimension was recorded for non-working people (PCS:77.1 ± 6.3; *p* < 0.001; ILQ:78.3 ± 9.3; *p* = 0.022). In the study group aged 30–39, a significantly higher QoL in all dimensions was recorded among men (*p* < 0.05). People with higher education rated QoL significantly better in PCS (73.3 ± 8.1; *p* = 0.008), MCS (79.6 ± 11.9; *p* = 0.002), and ILQ (76.5 ± 8.9; *p* = 0.001). People in relationships rated QoL significantly better in each of the dimensions compared to singles (respectively PCS:73.2 ± 8.2, *p* = 0.002; MCS:79.2 ± 12.2, *p* = 0.004; ILQ:76.2 ± 9.1, *p* = 0.001). Non-smokers rated QoL significantly better in MCS (78.8 ± 13.3; *p* = 0.026), and ILQ (75.8 ± 9.7; *p* = 0.021). In the study group aged 40–49, a significantly better level of QoL in each of the dimensions was recorded in the group of men (*p* < 0.05) and among the respondents with higher education (*p* < 0.05). Employment status also significantly affected QoL in each of the dimensions (*p* < 0.001). There was a significantly higher QoL in PCs (69.7 ± 10.5) and ILQ (70.8 ± 11.8) in working people and a significantly higher QoL level in MCS (72.5 ± 16.0) in non-working people. In the study group aged 50–59 years, higher education of the respondents (*p* < 0.001), remaining in the employment relationship (*p* < 0.001) and remaining in the relationship (*p* < 0.05) determined a significantly higher QoL in all dimensions of SF-36. In the study group aged 60–69 years, a better QoL in PCs was recorded among men (60.3 ± 13.2; *p* = 0.017). Higher education (*p* < 0.001) and the status of the working person (*p* < 0.001) significantly determined the higher level of QoL felt. Non-smokers indicated a significantly higher QoL level in the MCS dimension (61.9 ± 16.9; *p* = 0.044). In the study group aged 70–79 years, a better quality of life in all dimensions of SF-36 was recorded in the group of men (*p* < 0.05), among people living in the city (*p* < 0.05) and among people with higher education (*p* < 0.001). In the group of people ≥80 years of age in terms of MCS and ILQ, a significantly higher quality of life was demonstrated in the group of women (respectively: MCS:50.3 ± 17.2; *p* = 0.021; ILQ:47.0 ± 13.8; *p* = 0.019). In the PCS dimension, a significantly higher QoL level was recorded among people with higher education (47.3 ± 15.6; *p* = 0.014), in the retired group (43.0 ± 13.3; *p* = 0.036) and in the smoking group (49.2 ± 13.9; *p* = 0.009). The overall ILQ index was significantly higher among people with higher education (50.0 ± 14.9; *p* = 0.022) and smokers (51.4 ± 12.3, *p* = 0.028). Practising physical activity significantly determined a higher quality of life in almost all dimensions of SF-36 in people over 30 years of age. No significant influence was shown only in the youngest age group (Tables 4, 5).

**Table 4:** Average level of the QoL in individual spheres, considering the intergroup averages and the differences between them.

Variable	Age group																							
	<30						30–39						40–49						50–59					
	PCS		MCS		ILQ		PCS		MCS		ILQ		PCS		MCS		ILQ		PCS		MCS		ILQ	
	X	SD	X	SD	X	SD	X	SD	X	SD	X	SD	X	SD	X	SD	X	SD	X	SD	X	SD	X	SD
Gender																								
Female	74.1	6.6	77.1	12.8	75.6	8.9	71.5	9.3	76.4	14.4	74.0	10.8	68.1	11.3	69.6	16.6	68.9	12.8	65.7	13.9	67.8	16.4	66.8	13.9
Male	72.3	9.0	76.4	15.0	74.4	11.0	73.4	7.5	79.6	12.2	76.5	8.6	70.1	10.3	73.6	14.4	71.8	11.5	66.1	11.9	69.7	16.6	67.9	13.3
I $\bar{x}$ _1_ − $\bar{x}$ _2_I	1.8	2.4	0.7	2.2	1.2	2.1	1.9	1.8	3.2	2.2	2.5	2.2	2.0	1.0	4.0	2.2	2.9	1.3	0.4	2.0	1.9	0.2	1.1	0.6
*p*	**0.049**		0.656		0.272		0.003		0.002		0.001		0.027		0.003		0.003		0.702		0.173		0.313	
Place of residence																								
City	71.7	9.2	74.3	14.4	73.0	11.0	72.7	8.1	78.4	13.1	75.5	9.5	69.7	10.9	72.6	15.0	71.1	11.9	66.5	12.8	69.2	16.7	67.9	13.7
Village	74.7	6.4	80.1	13.5	77.4	8.7	72.2	8.9	77.7	13.7	74.9	10.2	68.3	10.9	70.2	16.5	69.3	12.4	65.2	12.9	68.3	16.3	66.7	13.5
I $\bar{x}$ _1_ − $\bar{x}$ _2_I	3.0	2.8	5.8	0.9	4.4	2.3	0.5	0.8	0.7	0.6	0.6	0.7	1.4	0.0	2.4	1.5	1.8	0.5	1.3	0.1	0.9	0.4	1.2	0.2
*p*	<0.001		<0.001		<0.001		0.397		0.513		0.417		0.119		0.074		0.064		0.234		0.488		0.325	
Education																								
Higher	72.8	8.0	76.7	12.9	74.8	9.4	73.3	8.1	79.6	11.9	76.5	8.9	70.7	10.0	73.3	15.1	72.0	11.4	68.9	11.2	73.7	14.9	71.3	12.1
Secondary	73.4	8.1	76.5	15.5	75.0	10.9	71.5	8.4	76.1	14.5	73.8	10.2	67.8	11.3	70.0	16.1	68.9	12.6	64.2	13.3	66.4	16.8	65.3	13.9
Elementary	68.2	14.7	74.7	22.4	71.5	18.4	69.8	12.9	74.9	18.4	72.3	14.8	65.4	12.4	68.1	16.4	66.7	13.4	64.3	14.4	64.3	16.7	64.3	14.0
I $\bar{x}$ _MAX_ − $\bar{x}$ _MIN_I	5.2	6.7	2.0	9.5	3.5	9.0	3.5	4.8	4.7	6.5	4.2	5.9	5.3	2.4	5.2	1.3	5.3	2.0	4.7	3.2	9.4	1.9	7.0	1.9
*p*	0.095		0.885		0.505		0.008		0.002		0.001		0.001		0.020		0.002		<0.001		<0.001		<0.001	
Employment status																								
Works	72.8	7.7	77.1	13.2	74.9	9.5	72.7	8.2	78.4	13.1	75.5	9.5	69.7	10.5	72.0	15.4	70.8	11.8	67.3	11.8	70.5	15.6	68.9	12.6
Disability pension	0.0	0.0	0.0	0.0	0.0	0.0	0.0	0.0	0.0	0.0	0.0	0.0	64.4	14.6	62.2	21.6	63.3	17.6	54.3	14.7	57.7	19.0	56.0	15.4
Retirement	0.0	0.0	0.0	0.0	0.0	0.0	0.0	0.0	0.0	0.0	0.0	0.0	53.4	9.6	54.3	14.7	53.9	11.1	58.8	14.4	58.8	17.6	58.8	14.9
Does not work	77.1	6.3	79.6	14.8	78.3	9.3	71.6	8.4	75.9	12.6	73.7	9.1	67.9	12.1	72.5	16.0	70.2	11.8	64.1	15.9	64.3	17.7	64.2	15.6
I $\bar{x}$ _MAX_ − $\bar{x}$ _MIN_I	4.3	1.4	2.5	1.6	3.4	0.2	1.1	0.2	2.5	0.5	1.8	0.4	16.3	5.0	18.2	6.9	16.9	6.5	13	4.1	12.8	3.4	12.9	3.0
*p*	<0.001		0.276		0.022		0.432		0.250		0.089		<0.001		<0.001		<0.001		<0.001		<0.001		<0.001	
Relationship																								
In the relationship	72.4	8.6	77.9	12.3	75.2	9.8	73.2	8.2	79.2	12.2	76.2	9.1	68.7	11.0	71.2	15.6	69.9	12.1	66.5	12.8	69.4	16.1	68.0	13.4
Single	73.2	8.2	75.9	15.2	74.5	10.7	71.2	8.8	76.1	15.1	73.6	10.7	71.3	10.3	73.2	16.4	72.3	12.4	63.1	13.1	65.6	18.0	64.3	14.5
I $\bar{x}$ _1_ − $\bar{x}$ _2_I	0.8	0.4	2.0	2.9	0.7	0.9	2.0	0.6	3.1	2.9	2.6	1.6	2.6	0.7	2.0	0.8	2.4	0.3	3.4	0.3	3.8	1.9	3.7	1.1
*p*	0.365		0.179		0.575		0.002		0.004		0.001		0.043		0.275		0.108		0.012		0.031		0.013	
Smoking																								
Yes	71.7	9.9	76.3	16.3	74.0	12.6	71.5	8.3	76.1	13.4	73.8	10.0	69.1	10.7	69.5	15.7	69.3	12.2	66.4	11.7	69.7	16.1	68.0	12.7
No	73.3	7.7	76.7	13.6	75.0	9.5	72.8	8.5	78.8	13.3	75.8	9.7	69.1	11.0	72.1	15.7	70.6	12.2	65.8	13.2	68.6	16.7	67.2	13.9
I $\bar{x}$ _1_ − $\bar{x}$ _2_I	1.6	2.2	0.4	2.7	1.0	3.1	1.3	0.2	2.7	0.1	2.0	0.3	0.0	0.3	2.6	0.0	1.3	0.0	0.6	1.5	1.1	0.6	0.8	1.2
*p*	0.081		0.794		0.377		0.065		0.026		0.021		0.909		0.990		0.100		0.654		0.504		0.537	
Physical Activity																								
Yes	72.9	8.5	77.0	14.1	75.0	10.5	74.3	6.7	81.0	11.7	77.7	8.0	72.1	9.2	75.3	14.2	73.7	10.5	69.2	11.6	73.5	14.5	71.3	12.0
No	72.9	8.2	76.1	14.6	74.5	10.3	70.4	9.7	74.7	14.3	72.6	10.9	66.8	11.5	68.6	16.2	67.7	12.7	63.9	13.1	65.8	17.1	64.8	13.9
I $\bar{x}$ _1_ − $\bar{x}$ _2_I	0.0	0.3	0.9	0.5	0.5	0.2	3.9	3.0	6.3	2.6	5.1	2.9	5.3	2.3	6.7	2.0	6.0	2.2	5.3	1.5	7.7	2.6	6.5	1.9
*p*	0.957		0.509		0.665		<0.001		<0.001		<0.001		<0.001		<0.001		<0.001		<0.001		<0.001		<0.001	

*X, average; SD, standard deviation; *p*-level of statistical significance; *t*-student test; $\bar{x}$ _1_ − $\bar{x}$ _2_, point difference between groups for the two analysed groups (value in points); I $\bar{x}$ _MAX_ − $\bar{x}$ _MIN_ I, point difference between the highest and the lowest value (value in percent); MCS, mental component summary; PCS, physical component summary; ILQ SF-36, index of life quality.

**Table 5:** Average level of the QoL in individual spheres, considering the intergroup averages and the differences between them.

Variable	Age group																	
	60–69						70–79						≥80					
	PCS	MCS	ILQ	PCS	MCS	ILQ	PCS	MCS	ILQ
	X	SD	X	SD	X	SD	X	SD	X	SD	X	SD	X	SD	X	SD	X	SD
Gender																		
Female	57.6	12.8	61.1	16.6	59.3	13.3	50.5	14.3	53.0	18.7	51.8	15.2	43.7	13.3	50.3	17.2	47.0	13.8
Male	60.3	13.2	61.5	17.2	60.9	14.0	53.4	15.8	57.8	18.7	55.6	16.3	40.0	13.3	44.6	15.7	42.3	13.5
I $\bar{x}$ _1_ − $\bar{x}$ _2_I	2.7	0.4	0.4	0.6	1.6	0.7	2.9	1.5	4.8	0.0	3.8	1.1	3.7	0	5.7	1.5	4.7	0.3
*p*	0.017		0.799		0.197		0.036		0.005		0.008		0.056		0.021		0.019	
Place of residence																		
City	59.8	12.6	62.1	17.1	60.9	13.7	53.4	15.1	56.9	19.4	55.2	16.1	42.7	14.1	48.2	17.0	45.5	14.4
Village	58.1	13.5	60.4	16.6	59.2	13.6	49.5	14.5	52.6	17.8	51.0	15.0	42.5	12.5	49.1	16.9	45.8	13.2
I $\bar{x}$ _1_ − $\bar{x}$ _2_I	1.7	0.9	1.7	0.5	1.7	0.1	3.9	0.6	4.3	1.6	4.2	1.1	0.2	1.6	0.9	0.1	0.3	1.2
*p*	0.150		0.239		0.157		0.003		0.010		0.003		0.903		0.686		0.851	
Education																		
Higher	62.8	12.2	66.1	17.1	64.5	13.7	58.5	14.0	64.5	19.0	61.5	15.5	47.3	15.6	52.7	16.8	50.0	14.9
Secondary	59.3	12.6	60.5	16.4	59.9	13.1	53.5	14.2	55.8	18.3	54.6	15.1	44.5	13.5	50.7	18.4	47.6	14.8
Elementary	53.9	13.8	58.1	17.2	56.0	14.0	46.9	14.7	50.3	17.9	48.6	15.1	40.2	12.4	46.3	15.5	43.3	12.5
I $\bar{x}$ _MAX_ − $\bar{x}$ _MIN_I	8.9	1.6	8.0	0.8	8.5	0.9	11.6	0.7	14.2	1.1	12.9	0.4	7.1	3.2	6.4	2.9	6.7	2.4
*p*	<0.001		0.001		<0.001		<0.001		0.001		<0.001		0.014		0.084		0.022	
Employment status																		
Works	62.8	11.8	65.1	17.0	64.0	12.9	55.6	18.3	58.0	21.3	56.8	19.7	0.0	0.0	0.0	0.0	0.0	0.0
Disability pension	55.1	13.6	54.1	16.9	54.6	13.7	49.4	14.3	50.3	14.8	49.9	12.8	29.9	4.76	45.3	9.79	37.6	5.19
Retirement	56.5	13.0	58.8	15.9	57.7	13.4	51.9	14.9	55.4	19.0	53.7	15.9	43.0	13.3	48.7	17.2	45.8	13.9
Does not work	58.7	14.3	64.5	16.1	61.6	13.1	44.4	14.9	49.0	16.8	46.7	15.0	44.3	15.1	50.5	16.7	47.4	14.8
I $\bar{x}$ _MAX_ − $\bar{x}$ _MIN_I	7.7	2.5	11.0	1.1	9.4	0.8	11.2	4.0	9.0	2.3	6.9	6.9	14.4	10.34	5.2	7.41	9.8	9.61
*p*	<0.001		<0.001		<0.001		0.272		0.332		0.265		0.036		0.828		0.282	
Relationship																		
In the relationship	59.0	13.0	61.8	16.8	60.4	13.6	51.5	14.5	54.4	18.7	52.9	15.4	43.1	14.3	48.6	18.1	45.8	14.7
Single	58.8	13.6	59.2	17.0	59.0	13.9	52.1	15.8	56.2	19.1	54.2	16.5	42.1	12.3	48.8	15.7	45.4	12.8
I $\bar{x}$ _1_ − $\bar{x}$ _2_I	0.2	0.6	2.6	0.2	1.4	0.3	0.6	1.3	1.8	0.4	1.3	1.1	1.0	2.0	0.2	2.4	0.4	1.9
*p*	0.892		0.141		0.329		0.648		0.334		0.428		0.580		0.919		0.838	
Smoking																		
Yes	57.3	13.1	57.8	16.6	57.5	13.6	48.8	15.2	52.3	19.0	50.6	16.1	49.2	13.9	53.5	13.5	51.4	12.3
No	59.3	13.1	61.9	16.9	60.6	13.6	52.2	14.8	55.4	18.7	53.8	15.6	41.8	13.1	48.1	17.3	45.0	13.9
I $\bar{x}$ _1_ − $\bar{x}$ _2_I	2.0	0.0	4.1	0.3	3.1	0.0	3.4	0.4	3.1	0.3	3.2	0.5	7.4	0.8	5.4	3.8	6.4	1.6
*p*	0.190		0.044		0.061		0.085		0.205		0.115		0.009		0.130		0.028	
Physical activity																		
Yes	61.1	12.5	62.8	16.5	61.9	13.3	58.6	14.2	62.8	19.2	60.7	15.6	50.2	14.8	60.6	15.7	55.4	14.3
No	57.8	13.2	60.3	17.0	59.0	13.7	49.2	14.4	52.2	17.9	50.7	15.0	41.9	13.0	47.5	16.7	44.7	13.4
I $\bar{x}$ _1_ − $\bar{x}$ _2_I	3.3	0.7	2.5	0.5	2.9	0.4	9.4	0.2	10.6	1.3	10.0	0.6	8.3	1.8	13.1	1.0	10.7	0.9
*p*	0.005		0.101		0.019		<0.001		<0.001		<0.001		0.006		0.001		0.001	

*X, average; SD, standard deviation; *p*-level of statistical significance; *t*-student test; $\bar{x}$ _1_ − $\bar{x}$ _2_, point difference between groups for the two analysed groups (value in points); I $\bar{x}$ _MAX_ − $\bar{x}$ _MIN_ I, point difference between the highest and the lowest value (value in percent); MCS, mental component summary; PCS, physical component summary; ILQ SF-36, index of life quality.

The value of the coefficient in each of the analysed pairs of variables indicates a significant dependence, both in the case of correlation with age (a) (*p* < 0.001) as a quantitative variable and with age groups (b) (*p* < 0.001). The exception is the lack of a significant correlation between the VT sphere and the age group (*p* = 0.771). The coefficient for the pair of variables SF-36 and age in most pairs of variables assumed a value of not less than 0.21 (MH sphere), except for the VT sphere (*r* = −0.05), and not more than −0.62 (PF sphere). A strong correlation (0.5 > *r* > 0.7) between age and the SF-36 spheres was noted in the following spheres: PF, RP, PCS, and ILQ. In the case of the S-36 correlation and age groups, the value of the coefficient indicated a very weak correlation (Table 6).

**Table 6:** Correlation coefficients between the SF-36 and age (quantitative variable) and age groups (qualitative variable).

Spheres	Age^a^	*p*	Age group^b^	*p*
PF	−0.62	<0.001	−0.26	<0.001
RP	−0.51	<0.001	−0.25	<0.001
BP	−0.50	<0.001	−0.26	<0.001
GH	−0.25	<0.001	−0.10	<0.001
VT	−0.05	0.003	0.01	0.771
SF	−0.33	<0.001	−0.18	<0.001
RE	−0.41	<0.001	−0.23	<0.001
MH	0.21	<0.001	0.14	<0.001
PCS	−0.60	<0.001	−0.27	<0.001
MCS	−0.50	<0.001	−0.28	<0.001
ILQ	−0.58	<0.001	−0.29	<0.001

*PF, physical functioning; RP, role physical; RE, role emotional; VT, vitality; MH, mental health; SF, social functioning; BP, bodility pain; GH, general health; MCS, mental component summary; PCS, physical component summary; ILQ SF-36, index of life quality.

The average differences between the analysed age groups were covered by the analysis. The analysis showed significant differences between 7 age groups (*p* < 0.001). Intergroup analyses were carried out. Significant differences in the comparative analysis between individual age groups were most often indicated. The most insignificant intergroup differences were indicated in the VT sphere. The exact data are contained in Tables 7, 8.

**Table 7:** Intergroup comparisons and the mean value of the difference between the compared groups.

Spheres	Age groups [mean percent difference beetwen age groups]											^a^*p*
	1 vs. 2	1 vs. 3	1 vs. 4	1 vs. 5	1 vs. 6	1 vs. 7	2 vs. 3	2 vs. 4	2 vs. 5	2 vs. 6	2 vs. 7	
PF	−0.30 ns	4.44**	8.81**	18.27**	32.47**	46.27**	4.74**	9.12**	18.58**	32.78**	46.58**	<0.001
RP	−2.14 ns	7.31**	15.94**	35.08**	47.34**	67.24**	9.45**	18.08**	37.22**	49.48**	69.39**	<0.001
BP	−0.55 ns	7.66**	13.83**	20.74**	27.30**	36.79**	8.21**	14.39**	21.30**	27.86**	37.34**	<0.001
GH	1.72*	2.79**	3.44**	4.11**	8.59**	10.83**	1.08 ns	1.72*	2.40**	6.87**	9.12**	<0.001
VT	−0.26 ns	0.65 ns	−0.82 ns	0.39 ns	0.44 ns	2.00**	0.91 ns	−0.56 ns	0.65 ns	0.70 ns	2.26**	<0.001
SF	−1.47 ns	5.28*	5.88**	13.00**	19.58**	24.16**	6.75**	7.35**	14.47**	21.05**	25.64**	<0.001
RE	−3.68*	6.84*	12.21**	27.18**	39.26**	48.82**	10.53**	15.90**	30.87**	42.94**	52.50**	<0.001
MH	2.33**	0.77 ns	−0.18 ns	−1.80*	−3.43**	−3.21**	−1.56*	−2.52**	−4.13**	−5.76**	−5.54**	<0.001
PCS	0.40 ns	3.83**	7.00**	13.92**	21.24**	30.29**	3.43**	6.60**	13.52**	20.84**	29.88**	<0.001
MCS	−1.49 ns	5.12**	7.78**	15.33**	21.64**	27.94**	6.60**	9.27**	16.82**	23.14**	29.43**	<0.001
ILQ	−0.55 ns	4.47**	7.39**	14.62*	21.44**	29.11**	5.01**	7.93**	15.17**	21.99**	29.66**	<0.001

*PF, physical functioning; RP, role physical; RE, role emotional; VT, vitality; MH, mental health; SF, social functioning; BP, bodility pain; GH, general health; MCS, mental component summary; PCS, physical component summary; ILQ SF-36, index of life quality; Age groups: 1–<30 years; 2–30–39 years; 3–40–49 years; 4–50–59 years; 5–60–69 years; 6–70–79 years; 7 > 80 years; *−*p* < 0.05 and **−*p* < 0.001 value of *p* for multiple comparisons; ns, no statistical correlation for multiple comparisons.

a *p*, *p*-level of statistical significance, *t*-student test, analysis between age groups.

**Table 8:** Intergroup comparisons and the mean value of the difference between the compared groups cont.

Spheres	Age groups [mean percent difference beetwen age groups]										^a^*p*
	3 vs. 4	3 vs. 5	3 vs. 6	3 vs. 7	4 vs. 5	4 vs. 6	4 vs. 7	5 vs. 6	5 vs. 7	6 vs. 7	
PF	4.38**	13.83**	28.03**	41.83**	9.46**	23.65**	37.45**	14.20**	28.00**	13.80**	<0.001
RP	8.63**	27.78**	40.03**	59.94**	19.15**	31.40**	51.31**	12.25**	32.16**	19.91**	<0.001
BP	6.18**	13.09**	19.65**	29.13**	6.91**	13.47**	22.95**	6.56**	16.04**	9.48**	<0.001
GH	0, 64 ns	1.32*	5.80**	8.04**	0.67 ns	5.15**	7.40**	4.48**	6.72**	2.24*	<0.001
VT	−1.47*	−0.26 ns	−0.21 ns	1.35 ns	1.21*	1.26*	2.82**	0.05 ns	1.61**	1.56*	<0.001
SF	0.60 ns	7.72**	14.30**	18.89**	7.12**	13.70**	18.28**	6.57**	11.16**	4.59*	<0.001
RE	5.37*	20.34**	32.41**	41.97**	14.97**	27.04**	36.60**	12.07**	21.64**	9.56*	<0.001
MH	−0.96 ns	−2.57**	−4.20**	−3.98**	−1.62*	−3.25**	−3.02**	−1.63**	−1.41**	0.22 ns	<0.001
PCS	3.17**	10.09**	17.41**	26.46**	6.91**	14.24**	23.28**	7.32**	16.37**	9.04**	<0.001
MCS	2.67*	10.22**	16.54**	22.84**	7.55**	13.87**	20.16**	6.31**	12.61**	6.30**	<0.001
ILQ	2.92**	10.16**	16.98**	24.65**	7.23**	14.05**	21.72**	6.82**	14.49**	7.67**	<0.001

*PF, physical functioning; RP, role physical; RE, role emotional; VT, vitality; MH, mental health; SF, social functioning; BP, bodility pain; GH, general health; MCS, mental component summary; PCS, physical component summary; ILQ SF-36, index of life quality; Age groups: 1–<30 years; 2–30–39 years; 3–40–49 years; 4–50–59 years; 5–60–69 years; 6–70–79 years; 7 > 80 years; *−*p* < 0.001 and **−*p* < 0.05 – value of *p* for multiple comparisons; ns, no statistical correlation for multiple comparisons.

a *p*, *p*-level of statistical significance, *t*-student test, analysis between age groups.

The chance of developing a better quality of life in the group of respondents was analysed, considering age groups and individual sociodemographic factors. In the group of people under 30 years of age, there was a 4-fold higher chance of a better quality of life in the PCS dimension (OR = 4.51;Cl:1.42–14.26) and 2 times higher in MCS (OR = 2.22;Cl:1.24–3.99) among people with higher education. In the group of employed people, there is almost a 2-fold higher chance of a better quality of life in PCS (OR = 1.70;Cl:1.03–2.79). There was also a 9-fold higher chance of a better quality of life in ILQ among people in relationships and a 12-fold higher greater chance of a better quality of life in ILQ among people engaged in physical activity. In the group of people aged 30–39, there is a 3.5-fold higher chance of feeling a better quality of life in the PCS dimension in the group of men (OR = 3.65;Cl:1.13–11.79). In the MCS dimension, the chance of a better QoL is almost twice as high in the group of people with higher education (OR = 1.69;Cl:1.01–2.82). Among people engaged in physical activity, there was a 4-fold higher chance of a better QoL in PCS (OR = 4.19;Cl: 1.36–12.90), a 2-fold higher chance in MCS (OR = 2.54;Cl:1.31–4.90) and a 3-fold higher chance of a better quality of life in the ILQ dimension (OR = 3.28;Cl:1.36–7.92). In the group of people aged 40–49 years, there is almost a 2-fold higher chance of developing a better quality of life in the PCS dimension (OR = 1.85;Cl:1.17–2.94), MCS (1.57;Cl:1.06–2.30), and ILQ (OR = 1.54;Cl:1.01–2.34) in the group of people with higher education. Practising physical activity 3-fold increases the chance of a better quality of life in the PCS dimension (OR = 2.74;Cl:1.40–5.33), MCS (OR = 3.01;Cl:1.73–5.24), and ILQ (OR = 3.31;Cl:1.76–6.24). In the group of people aged 50–59 years, there is a 2-fold higher chance of a better level of quality of life in the PCS dimension (OR = 2.47; Cl:1.21–5.04) among men and in the PCS dimension (OR = 1.78;Cl: 1.24–2.56), MCS (OR = 2.05; Cl:1.45–2.91), and ILQ (OR = 1.77;Cl:1.23–2.55) among people with higher education. A higher chance of a better quality of life was recorded in the group of people living in the city (OR = 2.04;Cl:1.02–4.09). Workers had a slightly higher chance of a better quality of life in each of the dimensions. People in relationships had almost a 2-fold higher chance of a better quality of life in the MCS dimension (OR = 1.69;Cl:1.02–2.79) and ILQ (OR = 1.69;Cl:1.00–2.86). Practising physical activity determines a 2-fold higher chance of a better quality of life in the PCS dimension (OR = 2.51;Cl:1.51–4.18) and in ILQ (OR = 2.64;Cl:1.58–4.44) and a 4-fold higher chance of a better quality of life in the MCS dimension (OR = 3.76;Cl:2.22–6.38). In the group of people aged 60–69, there is almost a double chance of developing a better quality of life in the PCS dimension in the group of people living in urban areas (OR = 2.08;Cl:1.26–3.45), with higher education (OR = 1.90;Cl:1.41–2.56) and among physically active people (OR = 1.94;Cl:1.30–2.90). A slightly higher chance of a better quality of life in the ILQ dimension was recorded among women (OR = 2.34;Cl1.09–5.01), among people with higher education (OR = 1.37;Cl:1.03–1.82) and among working people (OR = 1.34;Cl:1.12–1.60). In the group of people aged 70–79 years, there was a higher chance of a better quality of life in the PCS dimension among people living in the city (OR = 2.18;Cl:1.31–3.65), a more than twice higher chance in the group of people with higher education (OR = 2.36;Cl:1.79–3.12) and almost 4-fold higher chance among people engaged in physical activity (OR = 3.71;Cl:2.40–5.73). In the MCS dimension, a slightly higher chance of a better quality of life is observed among people with higher education (OR = 1.89;Cl:1.45–2.48) and among physically active people (OR = 2.39;Cl:1.57–3.63). There was a nearly 3-fold higher chance of a better quality of life in the general ILQ index among physically active people (OR = 2.69;Cl:1.76–4.12). People with higher education have a slightly higher chance of feeling a better quality of life (OR = 2.10;Cl:1.60–2.77). In the group of people ≥80 years of age, there was a higher chance of developing a better quality of life in the PCS dimension in the group of people living in the city (OR = 2.43;Cl:1.02–5.83) and with higher education (OR = 1.68;Cl:1.08–2.60). In the MCS dimension, people practising physical activity had a 4-fold higher chance of a better quality of life (OR = 4.38;Cl:1.62–11.83). In the ILQ 2 dimension – people with higher education had a higher chance of a better quality of life (OR = 1.63;Cl:1.08–2.44). People working (OR = 0.28;Cl:0.09–0.92) and non-smokers (OR = 0.47;Cl:0.17–0.95) had a lower chance of feeling a better QoL. The exact data are presented in Table 9.

**Table 9:** Estimating the occurrence of a better QoL (PCS. MCS. ILQ) in individual age groups, taking into account sociodemographic factors.

Age group vs.	Factors						
	Gender	Place of residence	Education	Employment status	Marital status	Smoking	Practising physical activity
<30							
PCS							
OR	1.27	2.00	4.51	1.70	^a^	1.72	^a^
−95Cl	0.30	0.12	1.42	1.03	^a^	0.40	^a^
+95Cl	5.46	32.10	14.26	2.79	^a^	7.36	^a^
*p*	0.743	0.623	0.010	0.035	^a^	0.463	^a^
MCS							
OR	0.83	0.49	2.22	1.33	0.65	2.02	1.62
−95Cl	0.39	0.19	1.24	0.98	0.29	0.97	0.80
+95Cl	1.79	1.27	3.99	1.80	1.43	4.14	3.27
*p*	0.220	0.142	0.007	0.069	0.279	0.054	0.175
ILQ							
OR	2.61	1.50	2.17	1.51	9.24	1.06	12.26
−95Cl	0.798	0.11	0.81	0.94	1.96	0.28	1.55
+95Cl	8.73	19.69	5.82	2.42	43.56	4.07	97.28
*p*	0.119	0.76	0.123	0.083	0.005	0.927	0.017
30–39							
PCS							
OR	3.65	0.46	1.14	1.30	1.18	0.84	4.19
−95Cl	1.13	0.14	0.51	0.74	0.45	0.27	1.36
+95Cl	11.79	1.45	2.57	2.26	3.10	2.61	12.90
*p*	0.030	0.185	0.747	0.360	0.733	0.763	0.012
MCS							
OR	1.63	0.86	1.69	0.85	1.84	1.30	2.54
−95Cl	0.61	0.32	1.01	0.46	0.99	0.66	1.31
+95Cl	4.36	2.34	2.82	1.56	3.42	2.56	4.90
*p*	0.323	0.77	0.043	0.594	0.054	0.442	0.005
ILQ							
OR	0.43	2.90	1.44	1.02	1.61	1.09	3.28
−95Cl	0.11	0.76	0.75	0.53	0.73	0.45	1.36
+95Cl	1.62	11.05	2.77	1.98	3.56	2.67	7.92
*p*	0.212	0.119	0.274	0.929	0.232	0.847	0.008
40–49							
PCS							
OR	1.54	0.79	1.85	1.38	1.46	1.03	2.74
−95Cl	0.86	0.38	1.17	1.05	0.49	0.52	1.40
+95Cl	2.76	1.64	2.94	1.83	4.33	2.07	5.33
*p*	0.145	0.521	0.009	0.023	0.547	0.923	0.003
MCS							
OR	1.57	1.20	1.57	1.21	0.42	1.07	3.01
−95Cl	0.97	0.58	1.06	0.93	0.17	0.62	1.73
+95Cl	2.54	2.49	2.30	1.57	0.99	1.87	5.24
*p*	0.064	0.615	0.022	0.145	0.048	0.801	<0.001
ILQ							
OR	1.50	1.09	1.54	1.18	0.56	0.96	3.31
−95Cl	0.88	0.43	1.01	0.88	0.23	0.51	1.76
+95Cl	2.55	2.76	2.34	1.57	1.34	1.83	6.24
*p*	0.127	0.847	0.045	0.262	0.193	0.908	<0.001
50–59							
PCS							
OR	2.47	2.04	1.78	1.41	1.07	1.01	2.51
−95Cl	1.21	1.02	1.24	1.13	0.61	0.62	1.51
+95Cl	5.04	4.09	2.56	1.77	1.88	1.64	4.18
*p*	0.012	0.042	0.002	0.002	0.811	0.969	<0.001
MCS							
OR	0.99	1.21	2.05	1.42	1.69	1.01	3.76
−95Cl	0.91	0.60	1.45	1.14	1.02	0.58	2.22
+95Cl	1.10	2.42	2.91	1.76	2.79	1.77	6.38
*p*	0.980	0.595	<0.001	0.001	0.040	0.963	<0.001
ILQ							
OR	0.42	0.44	1.77	1.58	1.69	0.79	2.64
−95Cl	0.20	0.17	1.23	1.27	1.00	0.46	1.58
+95Cl	0.87	1.14	2.55	1.97	2.86	1.40	4.44
*p*	0.020	0.090	0.002	<0.001	0.048	0.432	<0.001
60–69							
PCS							
OR	0.90	2.08	1.90	1.40	1.04	1.61	1.94
−95Cl	0.55	1.26	1.41	1.17	0.66	0.99	1.30
+95Cl	1.46	3.45	2.56	1.68	1.64	2.06	2.90
*p*	0.667	0.004	<0.001	<0.001	0.854	0.052	0.001
MCS							
OR	0.57	1.51	1.31	1.16	1.11	1.54	1.13
−95Cl	0.29	0.80	0.99	0.97	0.72	0.96	0.77
+95Cl	1.10	2.85	1.74	1.38	1.72	2.49	1.66
*p*	0.091	0.197	0.062	0.093	0.653	0.074	0.516
ILQ							
OR	2.34	0.41	1.37	1.34	1.12	1.29	1.17
−95Cl	1.09	0.19	1.03	1.12	0.72	0.79	0.80
+95Cl	5.01	0.87	1.82	1.60	1.75	2.10	1.73
*p*	0.029	0.020	0.032	0.001	0.610	0.306	0.411
70–79							
PCS							
OR	1.19	2.18	2.36	1.31	0.76	1.27	3.71
−95Cl	0.72	1.31	1.79	0.81	0.52	0.76	2.40
+95Cl	2.00	3.65	3.12	2.12	1.11	2.12	5.73
*p*	0.484	0.003	<0.001	0.275	0.157	0.361	<0.001
MCS							
OR	0.78	1.65	1.89	0.93	0.73	0.93	2.39
−95Cl	0.37	0.81	1.45	0.57	0.50	0.55	1.57
+95Cl	1.64	3.37	2.48	1.53	1.07	1.57	3.63
*p*	0.516	0.164	<0.001	0.784	0.109	0.784	<0.001
ILQ							
OR	2.08	0.55	2.10	1.03	0.91	0.98	2.69
−95Cl	0.88	0.24	1.60	0.48	0.62	0.48	1.76
+95Cl	4.88	1.26	2.77	2.22	1.33	2.03	4.12
*p*	0.092	0.156	<0.001	0.929	0.635	0.963	<0.001
>80							
PCS							
OR	0.82	2.43	1.68	0.31	1.23	0.47	1.89
−95Cl	0.31	1.02	1.08	0.10	0.68	0.19	0.74
+95Cl	2.17	5.83	2.60	1.02	2.24	1.13	4.86
*p*	0.696	0.044	0.020	0.052	0.485	0.090	0.182
MCS							
OR	1.01	0.68	1.47	0.73	1.11	0.47	4.38
−95Cl	0.36	0.26	0.98	0.26	0.65	0.20	1.62
+95Cl	2.87	1.79	2.20	2.01	1.88	1.09	11.83
*p*	0.983	0.436	0.056	0.537	0.701	0.079	0.003
ILQ							
OR	0.67	0.63	1.63	0.28	1.58	0.47	3.97
−95Cl	0.19	0.19	1.08	0.09	0.92	0.17	1.53
+95Cl	2.40	2.05	2.44	0.92	2.72	0.95	10.33
*p*	0.534	0.440	0.018	0.035	0.095	0.036	0.004

*PCS. physical component summary; MCS. mental component summary; ILQ SF-36. index of life quality; OR. odds ratio; −95Cl/+95Cl. 95% confidence interaval; *p*-level of statistical significance Wald Chi2 test. logistic regression.

a No volatility, analysis not avaiable.

## 4. Discussion

It is important to assess the quality of life in the group of healthy people in different age groups, which would show which of the dimensions of quality of life are best assessed and relevant in each age group and what sociodemographic factors significantly shape QoL in age groups. It is worth analysing the way of perceiving the quality of life changing with age. The main aim of the study was to assess the quality of life of healthy people in different age groups, considering sociodemographic factors, during the COVID-19 pandemic. The announcement of the pandemic and the introduction of numerous restrictions and changes in everyday human functioning had an impact on the assessment of the quality of life, especially in the mental and social sphere, which is a crucial point in the analysis presenting the level of quality of life in each of the dimensions.

The introduction of restrictions and maintaining social distance on the examples of many countries, in particular China, Singapore, Japan and South Korea, confirmed the effective reduction of the spread of the virus and the sense of psychological stress (36). The studies show that both long-term and short-term feelings of stress can affect the sense of quality of life, unless preventive measures are introduced earlier (37). During the pandemic, higher levels of perceived stress were reported by women (38–40), which could have a significant impact on the level of quality of life, especially in the mental sphere. The analysis of individual spheres of quality of life showed the lowest perceived quality of life level in the sphere of mental health (MH) (*X* = 45.6;Cl: 45.3–45.8) for the entire studied group and in the MCS dimension (*X* = 47.9;Cl:47.6–48.3). The highest level of perceived quality of life in the MCS dimension was recorded in the group of people aged 30–39 (78.1 ± 13.3), and the lowest in the oldest age group ≥80 (48.7 ± 17.0). Such a relationship was noted in other studies of the authors, where the analysis of the SF-36 tool was presented according to the key of the Polish adaptation of the questionnaire. The quality of life with the use of SF-36 was assessed among people over 65 years of age. This analysis showed a decrease in the level of quality of life in subsequent analysed age groups, including the oldest age group over 80 years of age (41). A number of studies available in the literature confirm a significant decrease in the perceived level of quality of life in subsequent years of human life in various dimensions, both physical and mental (42). It is worth emphasizing that the study on the quality of life carried out during the COVID-19 pandemic in the population of Egypt indicated a worse quality of life in the group of women (43), which was also confirmed by the carried out study. Women between the ages of 30 and 79 reported worse quality of life, compared to the group of men, in each of the PCS, MCS and ILQ dimensions. At age 80 and above, it is noted that the quality of life of women is significantly better compared to the opposite gender.

The level of perceived quality of life is also influenced by the environment and the place where a person lives. People under the age of 30 living in rural areas significantly better rated the quality in each of the SF-36 dimensions (PCS:74.7 ± 6.4; MCS:80.1 ± 13.5; ILQ:77.4 ± 8.7) compared to their peers living in the city. Such a frequency was also recorded in the group of people over 80 years of age. The opposite trend was recorded among people aged 30–79, where people living in the city in each of the dimensions indicated a better quality of life compared to people living in the village. The perception of the quality of life related to the place of residence in the era of the COVID-19 pandemic may have been involved with the restrictions related to the reduction of the spread of the virus, which were introduced by subsequent countries, in the scope of restrictions on movement and maintaining social distance (44–47). In addition, it was emphasized that these restrictions, increasing the degree of human isolation, caused new health problems and exacerbated existing ones (48–50). A significant decrease in the quality of life in the group of unrelated people cannot be confirmed on the basis of the carried out studies.

Other studies showed that poor quality of life in the somatic domain of the WHOQOL-BREF questionnaire is associated with low physical activity and low level of education among people between 25 and 44 years of age (42). Higher level of education is usually strongly associated with positive pro-health behaviours (51). Previous studies confirm a significantly higher level of quality of life in PCS among the population of Switzerland (52), Sweden (53), Brazil (54), Spain (55), and Norway (56). The authors’ studies also confirmed a significantly higher quality of life in the PCS dimension in people with higher education aged 30–79, but also in other dimensions. The quality of life was also significantly higher in people engaged in physical activity. The literature confirms that the lack of physical activity significantly increases the risk of hypertension, dyslipidaemia, and insulin insensitivity, which may affect the quality of life (57–60). In own studies, practising physical activity often increased the chance of developing a better quality of life several times.

In addition, the status of the unemployed person has a negative impact on the quality of life and mental health of a person in the group of young people, according to the carried out studies (61, 62). The study carried out on the Chinese population confirmed that the level of quality of life is higher in the group of working people (63). The author’s studies show significant correlations between the level of quality of life and the employment status (*p* < 0.001) among the respondents up to 69 years of age. However, the observed trends slightly differ in individual age groups. In the group of young people <30 years of age, unemployed people declare a higher quality of life compared to people working both in the physical dimension (PCS) and in the mental dimension (MCS). Respondents aged 40–69 most often indicated a significantly higher level of quality of life in each of the dimensions compared to other compared groups, including the unemployed. People in each of the retirement age groups had significantly lower levels of quality of life in the following dimensions: PCS,MCS and ILQ. At this point, it is worth emphasizing the importance of working conditions that significantly affect the level of the perceived QoL in various aspects (64). Summing up the factors, both internal (personality) and external, related to the environment in which a person functions, there is a whole spectrum and each of them may significantly determinate the QoL. Factors that may lead to deterioration of the QoL in the future should be identified early. These factors appear already in the group of healthy people.

### 4.1. Implications of all the available evidence

The analysis of the QoL of healthy people is a crucial element of health promotion. The aim of the study is to learn about the factors that significantly determine the level of the perceived QoL in a given population in individual representative age groups of the Polish population. At this point, the variability of factors should be emphasized, and what follows, the need for a permanent analysis of the QoL of healthy people. Only constant verification of the importance of factors will allow to implement new solutions in the field of health promotion, health policy creation and effective policy of combating disorders and diseases in society in the future.

The obtained results may be also a basis for comparing the results of the studies and analyses on the quality of life of other authors, especially if the analyses concern sick people. The author’s study includes healthy people aged 18 and over, so the basis for verification of correlations and comparisons is universal. In addition, the study includes an analysis of factors such as gender, age, place of residence, education, civil status, employment status, smoking, and physical activity and their impact on quality of life.

### 4.2. Limitation

The authors assessed the QoL in a group of healthy people, considering the analysed sociodemographic factors, including age groups. The impact of other factors and dependencies has not been studied, so the study and analysis also have some limitations. In the case of the analysis of factors: smoking and physical activity, both variables were included as dichotomous variables in the analysis. In the case of the analysis of the impact of smoking on the quality of life, the study of “passive smokers” did not assess, as well as the amount of cigarettes smoked. The analysis was to assess only at this stage whether there is an impact of this factor on the general sense of quality of life, without a detailed analysis whether the amount of cigarettes smoked in a unit of time significantly determines the quality of life of the subjects. The same assumption was made in the case of the analysis of the impact of physical activity on the quality of life of the individual. The frequency and intensity of physical activity were not assessed, only an overall assessment of whether physical activity has a significant impact on the quality of life of healthy people. The condition for selecting the answer “yes” in the case of physical activity was to meet the who guidelines on physical activity, which were indicated in point 2.3. of this article.

In addition, the influence of other factors, in addition to the analysed factors, including personality factors, may significantly shape the level of the QoL. In future studies, it is worth including an element assessing the mental sphere more widely. Similarly, in the case of the analysis of employment status, chronic stress related to the work performed is a critical issue.

The study conducted in Poland may differ from the QoL assessment of healthy people living in other countries. The causes of such a phenomenon may be the result of a different culture, diet, society and social policy, civic support system, etc. However, comparisons of such populations may constitute an interesting future area of studies and conclusions.

## 5. Conclusion

In the healthy population of Poland, the level of quality of life decreases in all dimensions with age. Men are more likely than women to assess their health better. A higher level of education significantly contributed to a better quality of life in the physical dimension. The practising of physical activity and the lack of smoking habit determine a higher level of quality of life more often. Analyses of QoL and factors influencing it in the population of healthy people should be constantly monitored and the conclusions should be implemented in health promotion activities.

## Data availability statement

The raw data supporting the conclusions of this article will be made available by the authors, without undue reservation.

## Ethics statement

Approval of the study was obtained from the Commission on Ethics of Scientific Research of the University of Information Technology and Management in Rzeszow (2/2022). The patients/participants provided their written informed consent to participate in this study.

## Author contributions

MK-S and AK: conceptualization, methodology, and writing—original draft preparation. MK-S: formal analysis and data collection. All authors contributed to the article and approved the submitted version.

## Conflict of interest

The authors declare that the research was conducted in the absence of any commercial or financial relationships that could be construed as a potential conflict of interest.

## Publisher’s note

All claims expressed in this article are solely those of the authors and do not necessarily represent those of their affiliated organizations, or those of the publisher, the editors and the reviewers. Any product that may be evaluated in this article, or claim that may be made by its manufacturer, is not guaranteed or endorsed by the publisher.
